# Altered metabolites in the periaqueductal gray of COVID-19 patients experiencing headaches: a longitudinal MRS study

**DOI:** 10.3389/fneur.2023.1323290

**Published:** 2024-01-05

**Authors:** Ping Jin, Feng Cui, Luping Zhang

**Affiliations:** Hangzhou TCM Hospital Affiliated to Zhejiang Chinese Medical University, Hangzhou, Zhejiang Province, China

**Keywords:** COVID-19, headache, periaqueductal gray, magnetic resonance spectroscopy, metabolites

## Abstract

**Background:**

Headache is one of the most common symptoms of acute COVID-19 infection. However, its mechanisms remain poorly understood, and there is a lack of studies investigating changes in the periaqueductal gray (PAG) in COVID-19 patients exhibiting headaches.

**Purpose:**

The study aimed to explore the alterations in metabolites of the PAG pre- and post-COVID-19 infection in individuals who suffered from headaches during the acute phase of the disease using proton magnetic resonance spectroscopy (^1^H-MRS).

**Methods:**

Fifteen participants who experienced headaches during the acute phase of COVID-19 were recruited. All subjects underwent two proton magnetic resonance spectroscopy (^1^H-MRS) examinations focusing on the PAG before and after they were infected. Metabolite changes were assessed between the pre- and post-infection groups.

**Results:**

The combined glutamine and glutamate/total creatine ratio (Glx/tCr) was increased in the PAG following COVID-19 infection. The total choline/total creatine ratio (tCho/tCr) in the pre-infection group was negatively correlated with the duration of headache during the COVID-19 acute phase.

**Conclusion:**

The present study indicates that PAG plays a pivotal role in COVID-19 headaches, thereby supporting the involvement of trigeminovascular system activation in the pathophysiology of COVID-19 headaches.

## 1 Introduction

Headache is one of the most prevalent symptoms after infection with severe acute respiratory syndrome coronavirus 2 (SARS-CoV-2) infection. It had a high incidence during the acute phase of coronavirus disease 2019 (COVID-19), presenting in up to 50% of patients. Notably, it also emerges as a primary symptom observed in the post-COVID-19, with a prevalence of 10% (1). Interestingly, some patients without a history of previous headache-related disorders experience new-onset headaches following SARS-CoV-2 infection (2). The presence of headache in the acute phase of COVID-19 is associated with a higher prevalence of long-term post-COVID headache (3). In addition, headache is a disabling condition in patients with long COVID-19, leading to a lower quality of life and general fatigue (4, 5). Therefore, it is important to understand the pathophysiology of headache in SARS-CoV-2 infection to facilitate its prevention.

Despite the high frequency of headaches during COVID-19 infection, the exact mechanism by which SARS-CoV-2 can cause headaches remains incompletely understood. Various potential pathophysiological mechanisms of headache in COVID-19 patients have been proposed, such as direct viral invasion of the nervous system, cytokine release syndrome, innate immune response, cerebral bioelectrical dysfunction, and the activation of the trigeminovascular system (6–10). The periaqueductal gray (PAG), a key region of the endogenous analgesia system, exhibits pathological changes in different types of headaches (11–13). Therefore, it is reasonable to posit that the PAG may be involved in the pathophysiology of COVID-19 headaches. However, there is a dearth of research on the PAG in the context of COVID-19 headaches.

Proton magnetic resonance spectroscopy (^1^H-MRS) is a non-invasive method that allows the *in vivo* study of tissue metabolism by leveraging the magnetic properties of hydrogen atomic nuclei. It enables the measurement of various metabolites and neurotransmitters, facilitating the investigation of dysregulation in excitatory and inhibitory processes of the brain, altered energy metabolism, changes in neuronal function, variations in cell density, and so on (14, 15). The aim of the present study was to investigate the alterations in metabolites of PAG pre- and post-COVID-19 infection in individuals who suffered from headaches during the acute phase of the disease, utilizing ^1^H-MRS technology. Furthermore, due to the observed gender disparities in COVID-19 and the greater incidence of headaches in female patients (16–19), the current study selectively enrolled female subjects to mitigate potential sex-related biases and improve the accuracy of the findings.

## 2 Materials and methods

### 2.1 Study design and participants

The participants in this study were recruited from local universities. This study employed a longitudinal prospective design, with participants initially scanned before being infected prior to the pandemic. Subsequently, after the pandemic, they were invited back for a second scan. The inclusion criteria were as follows: (1) patients >18 years old; (2) had a positive molecular swab test for SARS-CoV-2; and (3) experienced headaches during the acute phase of COVID-19. The exclusion criteria were as follows: those who (1) had underlying psychiatric disorders; (2) were taking any drugs affecting the central nervous system; and (3) had MRI contraindications such as claustrophobia. Demographic and clinical characteristics, including age, body mass index (BMI), years of education, past medical history, Self-rating Anxiety Scale (SAS), and Self-rating Depression Scale (SDS) prior to infection were recorded. Post-infection clinical data, including symptoms during infection, the duration of headache during the acute phase of COVID-19, and SAS and SDS scores, were documented.

### 2.2 MR image and spectrum acquisition

**Figure 1:**
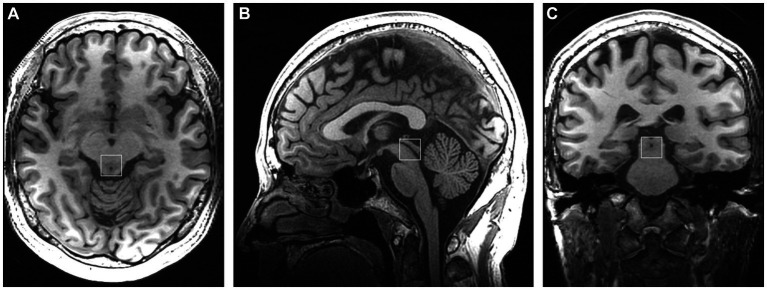
Placement of the single voxel in the axial **(A)**, sagittal **(B)**, and coronal **(C)** planes in the periaqueductal gray.

Subjects were scanned using a 3-Tesla GE Discovery MR750 scanner with a 32-channel head coil. Conventional T2-weighted images, T1-FLAIR, and DWI were obtained to ensure the absence of intracranial lesions. High-resolution T1-weighted scan (3D T1-BRAVO, TR = 8.2 ms, TE = 3.2 ms, flip angle = 12, matrix = 256 × 256, slice thickness = 1.0 mm with no gaps) was acquired for accurate voxel placement. For ^1^H-MRS, a volume of interest (VOI) was placed in the periaqueductal gray (Figure 1), and single-voxel point-resolved spectroscopy (PRESS) was employed with the following parameters: TR = 2000 ms, TE = 35 ms, voxel size = 15 × 15 × 15 mm^3^, total number of scans = 64, number of excitations (NEX) = 8, water suppressed, with automatic shimming.

### 2.3 MRS data processing

The MRS data were processed using the linear combination model (LCModel). Metabolites under investigation were creatine plus phosphocreatine (tCr), N-acetyl-aspartate (NAA), glycerophosphocholine and phosphocholine (total choline, tCho), myo-inositol (MI) and combined glutamine and glutamate (Glx). tCr serves as the reference metabolite and appeared stable in subjects. In this study, the relative concentration of metabolites (expressed as a ratio to tCr) was evaluated. Only metabolites with Cramer–Rao Lower Bounds (CRLB) smaller than 15% SD were considered reliable estimates and were included in further statistical analyses.

### 2.4 Statistical analysis

Statistical analyses were performed using the Statistical Package for Social Sciences software (SPSS 25.0). To assess the differences in metabolite ratios between post-infection and pre-infection data, as well as explore the relationship between metabolite ratios and clinical data, a paired *t*-test and Pearson’s correlation test were used for the data conforming to normal distribution. Meanwhile, the rank sum test and Spearman’s correlation test were used for non-normally distributed data. A *p*-value of less than 0.05 was considered statistically significant.

## 3 Results

### 3.1 Demographic and clinical characteristics

Initially, sixteen participants with COVID-19-related headaches were recruited for this study. Additionally, five participants who did not experience headaches during the acute phase were also included. One participant with COVID-19 headache and one without headache exhibited unsatisfactory spectral quality, with CRLB exceeding 15%, leading to their exclusion. Consequently, a total of fifteen participants with COVID-19 headaches and four participants without headaches were included in the final analysis. All participants had mild COVID-19 and did not require hospitalization. Demographic and clinical characteristics of the participants are summarized in Table 1. Four participants had a history of migraine before contracting COVID-19; however, they reported no migraine attacks during the infection period and had not received any prophylactic or medications before the scan, as detailed in Supplementary Table S2. There was no statistical difference in SAS and SDS scores among participants before and after infection (*p* > 0.05). The types and prevalence of COVID-19 symptoms are presented in Table 2.

**Table 1:** Demographic and clinical data of the patients (mean ± SD).

	COVID-19
Number of participants	15
Age (years)	23.67 ± 1.58
Education (years)	17.67 ± 1.67
Body mass index (kg/m^2^)	20.34 ± 1.79
SAS scores—before infection	30.93 ± 7.00
SDS scores—before infection	30.93 ± 7.07
SAS scores—after infection	29.27 ± 4.30
SDS scores—after infection	31.67 ± 7.49
Days between scan 1 and infection	35.47 ± 16.26
Days between infection and scan 2	59.67 ± 9.72
Days between scan 1 and scan 2	95.13 ± 16.84
Duration of symptoms (days)	8.53 ± 4.59
Duration of headache (days)	2.60 ± 0.91

SAS, self-rating anxiety scale; SDS, self-rating depression scale; scan 1, magnetic resonance imaging scanned before infection; scan 2, magnetic resonance imaging scanned after infection.

**Table 2:** COVID-19 symptoms during the acute phase.

Symptoms	*n* (%)^a^
Headache	15 (100.0)
Fever	12 (80.0)
Cough	11 (73.3)
Fatigue	13 (86.6)
Muscular pain	10 (66.6)
Sore throat	10 (66.6)
Nasal obstruction	8 (53.3)
Diarrhea	1 (0.1)
Conjunctivitis	0 (0)
Decreased appetite	8 (53.3)
Hypogeusia	4 (26.6)
Hyposmia	6 (40.0)
Nausea/vomiting	3 (20.0)
Dyspnea	0 (0)
Chest pain	4 (26.6)

a COVID-19 symptoms were evaluated in 15 participants.

### 3.2 Metabolite changes and correlation results

**Figure 2:**
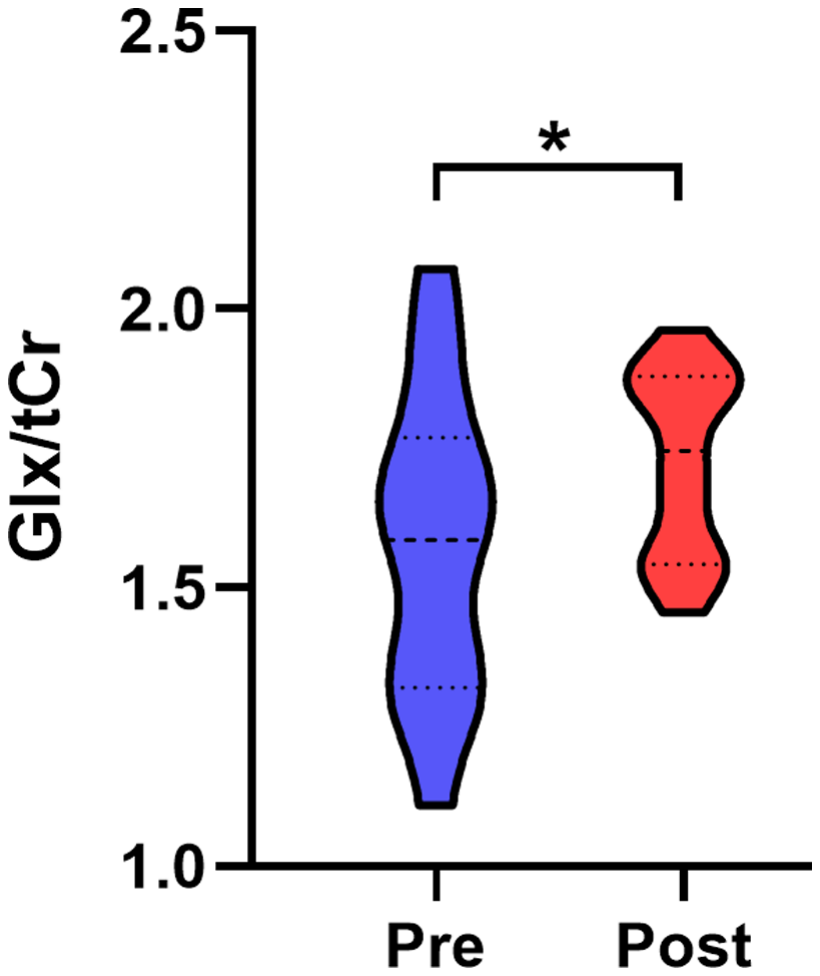
Comparison of PAG Glx/tCr ratio between post- and pre-infection groups. Glx/tCr ratio was significantly increased in the post-infection group relative to the pre-infection group. PAG, periaqueductal gray; Glx, combined glutamine and glutamate; tCr, creatine plus phosphocreatine; Pre, pre-infection group; Post, post-infection group; *, *p* < 0.05.

**Figure 3:**
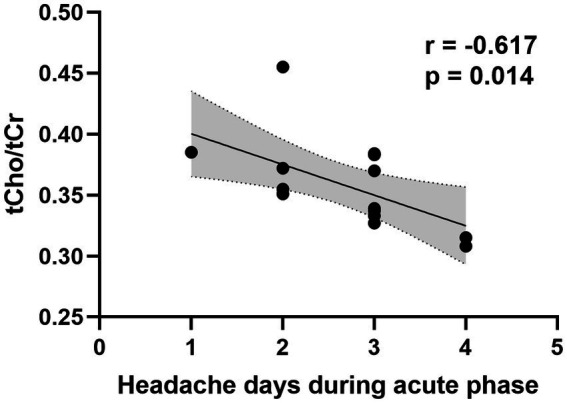
Correlation between PAG tCho/tCr ratio in the pre-infection group and duration of headache during the acute phase of COVID-19. PAG, periaqueductal gray; tCho, total choline; tCr, creatine plus phosphocreatine.

After infection, a significant elevation in Glx/tCr was observed in the PAG (*p* = 0.039) (Table 2 and Figure 2). However, this result was not observed in participants who did not experience headaches (Supplementary Table S1). There were no statistical differences in NAA/tCr, tCho/tCr, and MI/tCr between the two groups (all *p* > 0.05). The tCho/tCr in the pre-infection group was negatively correlated with the duration of headache during the acute phase of COVID-19 (*r* = −0.617, *p* = 0.014) (Figure 3).

## 4 Discussion

To the best of our knowledge, this study is the first to focus on PAG changes in patients who experienced COVID-19 headaches. The results revealed an elevated Glx/tCr ratio in the PAG following infection. Additionally, the tCho/tCr ratio in the pre-infection group was negatively correlated with the duration of headache during the acute phase of COVID-19.

PAG is an essential region of the endogenous analgesia system. Both take part in ascending and descending projections and modulate the ‘ON’ (facilitating the activity of neurons responding to noxious stimuli) and ‘OFF’ (ceasing firing immediately, inhibiting nociceptive responses) cells in the rostral ventromedial medulla to control nociceptive processing (20–22). Stimulation of the PAG can induce analgesic effects, while lesions in the PAG have been associated with causing headaches (13, 23). Glutamate and glutamine are typically quantified together as the glutamate/glutamine complex (Glx) due to their spectral overlap, making it challenging to separate their individual signals. Glutamate, the most abundant excitatory neurotransmitter in the brain, and glutamine, a precursor of glutamate, play a crucial role in signal transmission to and from the PAG (15). A glutamatergic pathway connecting the PAG with the rostral ventromedial medulla, specifically targeting OFF-cells, may exist (24). Activation of glutamatergic neurons in the PAG has been shown to suppress nociception, while inhibition of these neurons facilitates nociception (25). Therefore, the elevated Glx/tCr observed in the PAG after infection in our study may suggest an increased analgesic effect. Notably, no significant alteration in Glx/tCr was observed between pre- and post-infection in individuals who did not experience headaches. This finding may indicate a self-protective response against headaches. Moreover, PAG is involved in the descending control of trigeminovascular nociceptive traffic, with trigeminally evoked craniovascular pain being inhibited by PAG stimulation. Conversely, PAG activation has been observed following trigeminally evoked craniovascular pain (21, 22, 26, 27). As such, these results support the hypothesis that the pathophysiological mechanisms underlying headaches in COVID-19 patients may be linked to trigeminovascular activation.

The present study also unveiled a negative correlation between the tCho/tCr ratio in the pre-infection group and the duration of headaches during the acute phase of COVID-19. The tCho signal detected by MRS in the brain primarily originates from phosphocholine and glycerophosphocholine. Consequently, changes in the tCho signal reflect altered membrane turnover (increased membrane synthesis or breakdown) or changes in cell density, suggesting potential alterations in neuronal integrity and function (15). Therefore, the correlation results in this study suggest that the function of PAG before infection is related to the duration of headaches during the acute phase of COVID-19. Importantly, dysfunction in the PAG may contribute to prolonged headache duration. This discovery holds the potential to inform innovative clinical strategies for managing COVID-19-related headaches. Enhancing PAG function may reduce the likelihood and duration of headaches, thereby lowering the incidence of long COVID-19 headaches and improving patients’ overall quality of life.

The current study has certain limitations that warrant consideration. First, despite its longitudinal design, the sample size is relatively small, emphasizing the need for further validation with a larger cohort to enhance the generalizability of the results. Second, this study specifically focuses on patients who experienced headaches during the acute phase of COVID-19. Future research could extend its focus to individuals with long COVID-19 headaches to provide a more comprehensive understanding of the role of the PAG in this context. Third, it is important to note that four participants had a history of migraine before contracting COVID-19. While the longitudinal study design may mitigate the impact of migraine on the results to some extent, the potential influence of migraine on the outcomes cannot be completely disregarded.

## 5 Conclusion

To the best of our knowledge, this study represents the first exploration of PAG changes in patients with COVID-19 headaches. The findings underscore the significant role of the PAG in the pathophysiology of COVID-19 headaches. Notably, PAG activation post-infection appears to enhance the analgesic effect, and its pre-infection function correlates with the duration of headaches during the acute phase of COVID-19. Moreover, these results provide support for the hypothesis that trigeminovascular system activation contributes to the pathophysiology of COVID-19 headaches. These insights may pave the way for innovative approaches to the clinical management of COVID-19 headaches.

## Data availability statement

The original contributions presented in the study are included in the article/Supplementary material, further inquiries can be directed to the corresponding author.

## Ethics statement

The studies involving humans were approved by Ethics Committee of Hangzhou TCM Hospital Affiliated to Zhejiang Chinese Medical University. The studies were conducted in accordance with the local legislation and institutional requirements. The participants provided their written informed consent to participate in this study.

## Author contributions

PJ: Conceptualization, Data curation, Formal analysis, Funding acquisition, Investigation, Methodology, Validation, Visualization, Writing – original draft. FC: Data curation, Formal analysis, Methodology, Software, Writing – original draft. LZ: Conceptualization, Project administration, Resources, Supervision, Writing – review & editing.
