# Supplementary material for: Altered metabolites in the periaqueductal gray of COVID-19 patients experiencing headaches: a longitudinal MRS study
Source: Front Neurol. 2024 Jan 5;14:1323290. doi: 10.3389/fneur.2023.1323290 (PMC10796602; doi:10.3389/fneur.2023.1323290)
Supplement: Supplementary file 1 [file Data_Sheet_1.pdf]

Supplementary Table 1. Comparison of metabolite/total creatine ratios before and after infection in participants without headache (mean ± SD)

	Pre-infection (n=4)	Post-infection (n=4)	p-value
Glx/tCre	1.49±0.31	1.60±0.11	> 0.05
NAA/tCre	0.98±0.10	1.02±0.07	> 0.05
tCho/tCre	0.32±0.03	0.33±0.03	> 0.05
MI/tCre	1.16±0.11	1.07±0.18	> 0.05

Glx: combined glutamine and glutamate; NAA: N-acetyl-aspartate; tCho: total choline; MI: myo-inositol

Supplementary Table 2. The history of participants and the characteristics of COVID-19 headache.

	Suffered from other types of headaches prior to COVID-19 infection	Suffered headaches following COVID-19 infection	Characteristics of COVID-19 headache	VAS
Sub 01	N	N	heavy pain	4
Sub 02	N	N	heavy pain	6
Sub 03	N	N	dull pain	2
Sub 04	Migraine	N	dull pain	4
Sub 05	N	N	heavy pain	3
Sub 06	N	N	heavy pain	2
Sub 07	N	N	heavy pain	3
Sub 08	Migraine	N	heavy pain	4
Sub 09	Migraine	N	dull pain	2
Sub 10	N	N	heavy pain	3
Sub 11	Migraine	N	dull pain	5
Sub 12	N	N	heavy pain	3
Sub 13	N	N	heavy pain	1
Sub 14	N	N	sharp pain	3
Sub 15	N	N	dull pain	3

COVID-19: coronavirus disease 2019; VAS: visual analog scale; N: none.
